# Global Genomic Landscapes of *Lactiplantibacillus plantarum*: Universal GABA Biosynthetic Capacity with Strain-Level Functional Diversity

**DOI:** 10.3390/life16010047

**Published:** 2025-12-27

**Authors:** Monwadee Wonglapsuwan, Thitima Ninrat, Nattarika Chaichana, Thitaporn Dechathai, Sirikan Suwannasin, Kamonnut Singkhamanan, Rattanaruji Pomwised, Komwit Surachat

**Affiliations:** 1 Division of Biological Science, Faculty of Science, Prince of Songkla University, Songkhla 90110, Thailand; monwadee.wo@psu.ac.th (M.W.); rattanaruji.p@psu.ac.th (R.P.); 2 Department of Biomedical Sciences and Biomedical Engineering, Faculty of Medicine, Prince of Songkla University, Songkhla 90110, Thailand; thitimaninrat8@gmail.com (T.N.); naampueng.np@gmail.com (N.C.); oumthitaporn2543@gmail.com (T.D.); sirikan4036@gmail.com (S.S.); skamonnu@medicine.psu.ac.th (K.S.)

**Keywords:** *Lactiplantibacillus plantarum*, GABA, *gadB*/*gadC*, pangenome, accessory genome, plasmid, CAZy, bacteriocin, fermented foods, probiotics

## Abstract

*Lactiplantibacillus plantarum* is widely used in fermented foods and as a probiotic, yet the genomic basis underlying its γ-aminobutyric acid (GABA) production capacity and strain-level functional diversity remains incompletely resolved. We analyzed 1240 publicly available genomes to map species-wide genome architecture, the distribution of GABA-related genes, and accessory drivers of phenotypes. Pangenome analysis identified 45,201 gene families, including 622 strict core genes (1.38%) and 444 soft-core genes (2.36%). The accessory genome dominated (3138 shell and 40,997 cloud genes; 97.64%), indicating a strongly open pangenome. In contrast, the GABA (gad) operon was universally conserved: *gadB* (glutamate decarboxylase) and *gadC* (glutamate/GABA antiporter) were present in all genomes regardless of isolates source. Accessory-genome clustering revealed ecological and geographic structure without loss of the operon, suggesting that phenotypes variability relevant to fermentation and probiotic performance is primarily shaped by accessory modules. Accessory features included carbohydrate uptake and processing islands, bacteriocins and immunity systems, stress- and membrane-associated functions, and plasmid-encoded traits. Analysis of complete genomes confirmed substantial variation in plasmid load (median = 2; range = 0–17), highlighting the role of mobile elements in niche-specific adaptation. Carbohydrate-Active Enzymes database (CAZy) and biosynthetic gene cluster (BGC) profiling revealed a conserved enzymatic and metabolic backbone complemented by rare lineage-specific functions. Collectively, these results position *L. plantarum* as a genetically stable GABA producer with extensive accessory-encoded flexibility and provide a framework for rational strain selection.

## 1. Introduction

Gamma-aminobutyric acid (GABA) is a non-proteinogenic amino acid with antihypertensive, anxiolytic, and neuromodulatory benefits [1,2,3]. Increasing interest in functional foods has positioned GABA as a high-value bioactive metabolite, and microbial fermentation provides an efficient and sustainable route to its production [4].

Among LAB, *Lactiplantibacillus plantarum* is one of the most versatile and widely distributed lactic acid bacteria, occurring in diverse fermented foods, plant environments, animal hosts, and the human gut [5,6]. It has long been used in traditional fermentation processes and is recognized as a probiotic due to its acid and bile tolerance, vitamin biosynthesis, and production of antimicrobial peptides [7,8] Several strains of *L. plantarum* have been experimentally reported to produce high levels of GABA in foods such as kimchi, pickles, rice bran, cheese, and fermented fish [9,10,11,12,13,14,15]. However, the genomic basis for GABA biosynthesis has not yet been characterized at a global species-wide scale.

The glutamate decarboxylase (GAD) system—typically comprising the decarboxylase gene (*gadB* or *gadA*) and the glutamate/GABA antiporter (*gadC*)—is the primary pathway for GABA biosynthesis in bacteria [16,17]. Although this operon contributes to both acid resistance and GABA productivity, its conservation and genomic organization across hundreds of *L. plantarum* strains remain unclear. Phenotypic variation in GABA production suggests that additional accessory factors may modulate capacity [18].

Advances in comparative genomics and pangenome analysis now enable species-wide resolution of operon conservation and accessory gene diversity [19,20]. Previous genomic studies in Lactobacillaceae have investigated the presence and organization of GABA-related genes in selected strains or limited genome collections, primarily focusing on functional characterization or strain-level probiotic potential [21,22,23]. While these studies have provided important insights into the genetic basis of GABA biosynthesis, they have not addressed the extent of operon conservation, pangenome context, or accessory-genome contributions at a species-wide scale. In contrast, the present study analyzes 1240 *L. plantarum* genomes to systematically evaluate the conservation of GABA-related genes alongside pangenome structure, plasmid diversity, and functional accessory modules. This large-scale comparative framework enables a clearer distinction between core GABA biosynthetic capacity and lineage-specific genomic features that may modulate phenotypic performance in fermentation and probiotic applications.

In this study, we analyzed 1240 publicly available *L. plantarum* genomes to establish a comprehensive phylogenomic framework and to examine the distribution of GABA-related genes. Specifically, we sought to (i) determine the conservation and genomic organization of the GAD operon, (ii) evaluate plasmid content, secondary metabolite clusters, and carbohydrate-active enzymes, and (iii) characterize probiotic-associated marker genes and accessory genome diversity. By resolving both conserved and variable genomic features, our work provides new insights into the genetic foundations of *L. plantarum* as a robust GABA producer and highlights its potential applications in functional food development and probiotic innovation.

## 2. Materials and Methods

### 2.1. Genome Dataset Collection

A total of 1240 publicly available *L. plantarum* genomes were retrieved from the NCBI (https://www.ncbi.nlm.nih.gov/, accessed on 15 March 2025) RefSeq and GenBank databases. Metadata such as isolation source (Food, Human, Environment, Animal, or NA) and country of origin were curated from BioSample entries and manually cross-validated. To minimize artefactual inflation of gene families in the pangenome, we applied simple, transparent structural-quality filters to all NCBI assemblies. Only isolate genomes labelled as complete, chromosome, scaffold, or contig-level, were retained, while metagenome-assembled genomes (MAGs), environmental bins, and clearly misclassified entries were removed. Assemblies were further filtered to include only those with N50 ≥ 900 kb, ≤700 contigs, and genome sizes within the species-typical range (approximately 2.6–3.9 Mb). These criteria ensured exclusion of highly fragmented or atypical genomes while maintaining a broad and representative dataset (Table S1).

### 2.2. Genome Annotation

Each genome was reannotated with Prokka v1.14.6 [24] using default parameters to generate standardized GFF, GBK, and protein FASTA files. Functional annotation was supplemented with eggNOG-mapper v2 [25] and KEGG Ortholog assignment using KofamScan v1.3.0 (HMMER3 profiles, adaptive thresholds) [26].

### 2.3. Identification of GABA-Related Genes

All *L. plantarum* genome assemblies were compiled for the identification of GABA-related genes. Each genome was annotated with Prokka v1.14.6 [24] under default parameters to obtain per-strain tabular annotations and proteomes. KEGG Orthologs were assigned to the predicted proteins using KofamScan v1.3.0 [26] with HMMER3 profiles and adaptive score thresholds, retaining GABA-module KOs, particularly K01580 for *gadA*/*gadB*, when the built-in cut-off (E ≤ 1 × 10^−10^) was satisfied. To increase sensitivity across variably annotated loci, we supplemented KEGG-based identification with homology searches. Each proteome was queried against curated reference sets for *gadA*, *gadB*, *gadC*, *gadR*, *gabT*, *gabD*, *gabP*, *gltP*, and *gltT* using DIAMOND BLASTp v2.1 [27] (E ≤ 1 × 10^−10^; ≥35% identity; ≥50% coverage). Because *gadC* is frequently misannotated, we additionally used MMseqs2 v14 nucleotide-level searches [28] (E ≤ 1 × 10^−10^; ≥70% identity; ≥60% coverage) using a curated *gadC* query. A gene was considered present if supported by at least one evidence class (Prokka annotation, KO assignment, or homology hit). We generated binary presence–absence matrices and detailed homology statistics per gene and genome.

### 2.4. Plasmid Identification and Distribution

Plasmid content was assessed from complete-genome assemblies (n = 330) by counting the number of replicons in each FASTA file. The chromosome was distinguished from plasmids based on annotation metadata, with all additional replicons beyond the primary chromosome considered plasmids. The total plasmid count per genome was summarized across strains, and comparative distributions between isolation sources were tested using the Kruskal–Wallis test, followed by Holm-adjusted pairwise Mann–Whitney U tests.

### 2.5. Probiotic Marker Gene Analysis

Probiotic-associated genes (acid and bile tolerance, stress response, adhesion, bacteriocins, vitamin biosynthesis, and glutathione metabolism) were screened using DIAMOND BLASTp against curated reference sequences with the thresholds described above [27]. Results were summarized as prevalence percentages and visualized with radar plots using ggplot2 in R v4.3.

### 2.6. Secondary Metabolite Biosynthetic Gene Cluster (BGC) Analysis

BGCs were predicted using antiSMASH v7.0 [29] with all secondary metabolite detection modules enabled. Cluster categories such as Ribosomally synthesized and post-translationally modified peptides (RiPPs), non-ribosomal peptide synthetases (NRPS), polyketide synthases (PKS), terpenes, etc., were tabulated per genome, and prevalence statistics were summarized in bar plots.

### 2.7. Carbohydrate-Active Enzyme (CAZyme) Annotation

CAZymes were annotated using the dbCAN3 meta server [30], integrating three tools: HMMER against Carbohydrate-Active Enzymes database (CAZy) HMMdb (E ≤ 1 ×10^−15^, coverage ≥ 0.35), DIAMOND, and Hotpep. Families were assigned when supported by at least two tools. Family- and class-level distributions were calculated, and genome × CAZy-family matrices were clustered hierarchically.

### 2.8. Pangenome Analysis

A pan-genome was constructed using Roary v3.13 [31] with a 95% BLASTp identity cut-off. Core, soft-core, shell, and cloud gene families were defined as present in 99–100%, 95–99%, 15–95%, and <15% of genomes, respectively. Rarefaction curves were plotted to assess the openness of the pan-genome.

### 2.9. Phylogenomic Analysis

Accessory-genome presence–absence matrices were used to infer trees with FastTree v2.1.11 [32] under the Jaccard distance model. Phylogenies were visualized and annotated in iTOL v7 [33], with metadata overlays including gad operon presence/absence, isolation source, and country of origin.

### 2.10. Statistical Analysis

All statistical tests were performed in R v4.3. ECDF, box-and-swarm plots, and radar charts were generated using ggplot2. *p*-values < 0.05 after multiple testing correction were considered significant.

## 3. Results and Discussion

### 3.1. Genome Characteristics of Lactiplantibacillus plantarum

The analysis of 1240 *Lactiplantibacillus plantarum* assemblies revealed a highly conserved genomic architecture. Genome sizes ranged from 2.61 Mb to 3.90 Mb, with the majority of strains clustering tightly between 3.2 and 3.3 Mb. Gene counts were similarly consistent, varying from 2499 to 4169 predicted coding sequences, with most genomes containing ~3000–3300 genes. These findings indicate a stable genome size and coding capacity across the species, consistent with its metabolic versatility and adaptation to diverse niches [5,34].

Assembly quality varied substantially across datasets. Nearly half of the genomes were available only at the contig level, whereas 27.7% were scaffold-level and 26.7% were complete genomes. A small number (1.8%) were available as chromosome-level assemblies. These differences reflect the ongoing expansion of genomic resources and highlight the value of high-quality assemblies for analyses of traits such as GABA production, stress tolerance, and probiotic potential [35].

Among 330 complete genomes, 255 (77.3%) carried at least one plasmid, with a median of two plasmids per genome (IQR: 1–5; range: 0–17). Approximately half (50.9%) carried 1–4 plasmids, whereas 22.7% lacked plasmids entirely. Genomes encoding ≥10 plasmids were rare (3.3%), indicating substantial variation in plasmid-encoded accessory traits [36,37].

Plasmids in *L. plantarum* often encode carbohydrate-utilization pathways, stress-tolerance mechanisms, and antimicrobial factors [36,38,39]. The observed distribution therefore likely reflects niche-specific adaptation and domestication in fermented-food environments.

**Figure 1:**
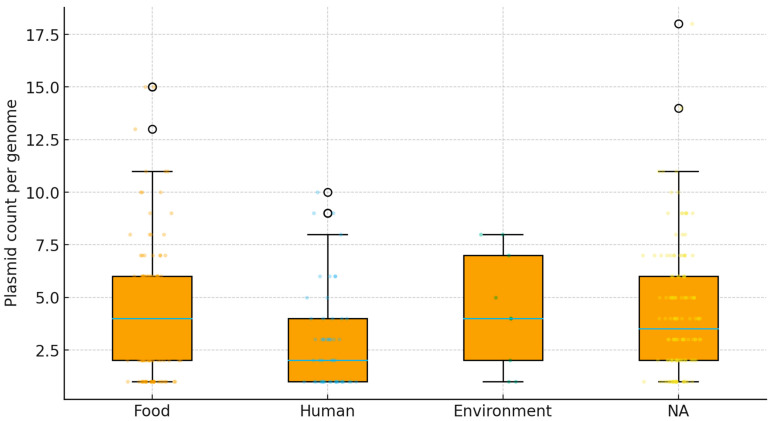
Plasmid load by isolation source. Box-and-swarm plot of plasmid counts per genome for Food (orange), Human (blue), Environment (green), and NA (yellow) categories. Boxes show the median and interquartile range; whiskers extend to 1.5 × IQR; points are individual genomes with horizontal jitter. Global differences were tested with Kruskal–Wallis, and pairwise Mann–Whitney tests with Holm correction are reported in the accompanying statistics file. “NA” denotes records without a source label or missing.

Food-associated strains tend to carry more plasmids than human-derived strains. A global test across sources was significant (Kruskal–Wallis, *p* = 0.028) as shown in Figure 1. Holm-adjusted pairwise tests showed food greater than human (*p* = 0.0167). The NA group (unresolved or other) also exceeded human (*p* = 0.035). The environment was intermediate and not significantly different from the food or the human after correction. ECDF curves for food isolates are shifted to the right, indicating more genomes with higher counts. A larger fraction of food isolates carries at least one plasmid and at least ten plasmids when compared to humans. Plasmids in Lactobacillaceae are well recognized as carriers of niche-adaptive and technologically relevant traits, and variation in plasmid burden may therefore reflect ecological or process-associated selection. For fermentation-associated origins, plasmid-encoded functions frequently include carbohydrate utilization modules (e.g., sugar transport/catabolic operons that enhance growth and acidification in defined substrates), antimicrobial competitiveness (bacteriocin-related loci), and phage defense systems that can improve robustness during industrial fermentations [40]. In contrast, environmental or plant-associated isolates may benefit from plasmid-borne stress-adaptation functions (e.g., oxidative or general stress tolerance), which have been reported in *L. plantarum* and related taxa [41]. Although our current analysis primarily quantified plasmid variability across sources, systematic annotation of plasmid gene content followed by source-enrichment testing would be a valuable next step to directly evaluate whether specific plasmid-borne functions track with environmental versus technological origins. Overall, plasmid load appears niche-linked; food-related contexts may favor plasmid maintenance and acquisition, consistent with functions for carbohydrate use, stress tolerance, and antimicrobial traits in fermented-food settings [40,42].

### 3.2. Conservation of GABA-Related Genes and Reported Strain Production

Analysis of 1240 *L. plantarum* genomes confirmed that the genetic capacity for γ-aminobutyric acid (GABA) biosynthesis is highly conserved (Table 1). Both *gadB* and *gadC* were universally present (100%). The glutamate transporter *gltT* was also nearly universal (99.8%). In contrast, catabolic genes (*gabT*, *gabP*, *gltP*) were absent, and *gabD* appeared only in three strains (0.24%). This suggests that *L. plantarum* relies almost exclusively on the *gadB*–*gadC* system for GABA biosynthesis, without conserved pathways for GABA catabolism.

**Figure 2:**
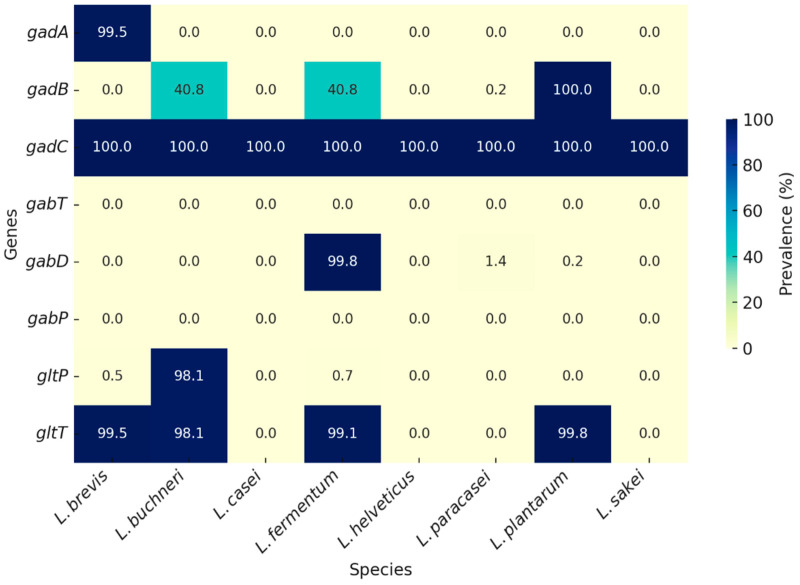
Comparative prevalence of γ-aminobutyric acid (GABA)-related genes across *Lactobacillus* species. Presence/absence analysis revealed that the glutamate/GABA antiporter (*gadC*) is universally conserved (100%) across all eight species. In *L. plantarum*, *gadB* (100%) was conserved together with *gadC* and *gltT* (99.8%), while catabolic genes such as *gabT* and *gabP* were absent and *gabD* was only sporadically present (0.24%). *L. brevis* exhibited high prevalence of *gadA* (99.5%) with *gadC* (100%) and *gltT* (99.5%), consistent with strong GABA-producing potential. *L. buchneri* and *L. fermentum* carried *gadB* at moderate prevalence (40.8%), with *L. fermentum* additionally encoding *gabD* (99.8%), indicating both biosynthetic and catabolic capacity. In contrast, *L. casei*, *L. helveticus*, and *L. sakei* harbored only *gadC* (100%), suggesting limited intrinsic GABA production. *L. paracasei* showed a unique intermediate profile with *gadB* present in a minority of strains (0.2%) alongside universal *gadC*. Together, these findings highlight species-specific genomic strategies for GABA metabolism, with *L. plantarum*, *L. brevis*, and *L. fermentum* standing out as the strongest candidates for functional food applications.

When compared with other *Lactobacillus* species, a clear interspecific variation emerges (Table 2 and Figure 2). *Levilactobacillus brevis* exhibited the most complete GABA-related gene repertoire, with nearly universal presence of *gadA* (99.5%), *gadC* (100%), and the transporters *gltT* (99.5%) and *gltP* (0.5%), consistent with its reputation as a strong GABA producer in fermented foods [43]. *Lentilactobacillus buchneri* showed moderate conservation, with *gadB* (57.4%), *gadC* (100%), and both *gltT/gltP* (98.1%), suggesting strain-dependent variability [44]. *Limosilactobacillus fermentum* demonstrated a unique profile, carrying *gadB* (40.8%), *gadC* (100%), *gabD* (99.8%), and *gltT* (99.1%), indicating both GABA biosynthetic and catabolic potential [45]. In contrast, *Lacticaseibacillus casei* and *Lactobacillus helveticus* carried only *gadC* (100%) without other biosynthetic or catabolic genes, suggesting limited intrinsic GABA production ability. Notably, *Lacticaseibacillus paracasei* displayed a unique intermediate pattern, with *gadC* (100%), low-level presence of *gadA* (0.2%), and *gabD* (1.4%), pointing to a restricted but potentially strain-specific GABA capacity [1,46].

These genomic patterns are consistent with experimental reports of high GABA production across diverse *L. plantarum* strains (Table 3). Multiple isolates from fermented foods, plant materials, and even insect sources have demonstrated substantial GABA yields. For instance, *L. plantarum* LSI2-1 from Thai fermented vegetables produced 22.94 g/L GABA, *L. plantarum* EJ2014 from rice bran yielded 19.8 g/L, and *L. plantarum* L10-11 from *Plaa-som* (fermented fish, Thailand) produced 15.74 g/L. Similarly, *L. plantarum* K154 from kimchi achieved 15.53 g/L, while other strains, such as *L. plantarum* BC114 (Sichuan *paocai*, 3.45 g/L) and *L. plantarum* FNCC 260 (Indonesian fermented foods, 0.809 g/L), also exhibited significant production. However, these published functional studies are important to emphasize that the present genome-based analysis remains predictive. The presence of *gad* genes indicates biosynthetic potential but does not guarantee enzymatic activity across all strains or conditions. Functional expressions of the GAD system are known to be regulated by environmental cues such as pH and glutamate availability. Therefore, experimental validation remains necessary to directly link gene presence with GABA production at the strain level.

Taken together, the universal conservation of *gadB* and *gadC* across all *L. plantarum* genomes [4], combined with extensive experimental evidence of high GABA production [9,43,44], highlights this species as a genetically stable and phenotypically robust GABA producer. Comparative analyses further emphasize that while several *Lactobacillus* species (*L. brevis*, *L. buchneri*, *L. fermentum*) harbor complementary GABA-related genes, *L. plantarum* stands out due to its genomic stability and extensive strain-level reports [1,57], underscoring its strong potential for application in functional foods and probiotic development.

### 3.3. Probiotic Marker Genes

**Figure 3:**
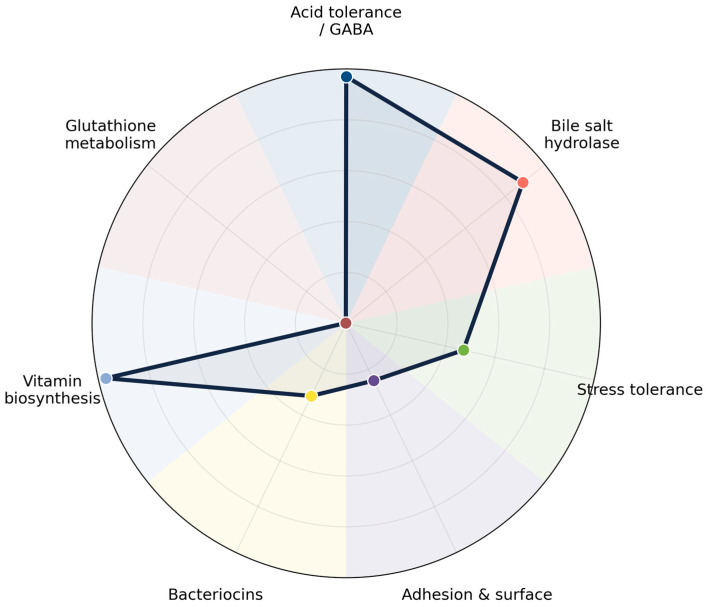
Radar plot illustrating the prevalence of probiotic-associated gene categories in *L. plantarum* (n = 1240 genomes). The polygon represents the mean prevalence of genes within each category, while vertical whiskers indicate the observed minimum and maximum prevalence values for individual genes. Colored markers denote the mean prevalence per category: acid tolerance/GABA metabolism (dark blue), vitamin biosynthesis (light blue), bile salt hydrolase (pink), stress tolerance (green), bacteriocins (yellow), adhesion and surface proteins (purple), and glutathione metabolism (brown). The plot highlights universally conserved traits (*gadB*, *gadC*, *gltT*; vitamin biosynthesis genes) alongside variable or strain-specific features such as stress tolerance, bacteriocin production, adhesion, and glutathione metabolism.

Analysis of 1240 genomes of *L. plantarum* revealed a highly conserved set of genes associated with probiotic functionality, as summarized in Table 4. The distribution of these genes across functional categories is further illustrated in the radar plot (Figure 3), which highlights both universally conserved and variable probiotic traits in the species.

The universal presence of *gadB*, *gadC*, and *gltT* highlights the strong conservation of acid-tolerance and GABA-production pathways [1,58,59]. These genes are central to the conversion of GABA, a metabolite that not only improves acid resistance but also provides neuroactive benefits when consumed [60,61]. Minor contributors to GABA metabolism (*gabD*, *gabT*) were rare, reinforcing the centrality of the *gadB*–*gadC* system [43,44]. This conserved genetic feature strongly supports the designation of *L. plantarum* as a key GABA-producing probiotic [15].

Genes linked to bile salt hydrolase activity (*bsh*) were detected in ~89% of genomes, consistent with enhanced survival in the gastrointestinal tract and potential cholesterol-lowering effects [62,63]. Stress-response genes (*dnaK*, *groEL*) were moderately conserved, indicating variation in thermotolerance and oxidative-stress resilience across strains [64,65]. Their uneven distribution suggests that some strains are inherently more robust under challenging conditions, an aspect of relevance for industrial fermentation and probiotic formulation.

Adhesion-related genes such as *slpA* were present in 25% of strains, implying strain-specific variation in host-interaction capacity [66,67]. Bacteriocin genes (*plnE*, *plnF*) showed ~47% prevalence, whereas pediocin-like genes were rare (~1%), suggesting heterogeneous antimicrobial potential [68,69]. Notably, bacteriocin-related genes showed an irregular and strain-dependent distribution, indicating that antimicrobial activity is not a universal trait in *L. plantarum* [70]. This heterogeneity highlights bacteriocin gene content as a valuable criterion for targeted strain selection when antimicrobial functionality is desired, particularly for food preservation or pathogen control applications. A defining feature of *L. plantarum* is its universal capacity for vitamin biosynthesis. Genes responsible for folate (*folA–C*) and riboflavin (*ribA–E*) biosynthesis were identified in all genomes (100% prevalence). This consistent genetic trait highlights the species’ role as a nutritional enhancer in fermented foods, capable of contributing essential B-vitamins to the diet [71,72].

In contrast, glutathione metabolism genes (*gshA*, *gshB*) were nearly absent (<0.5%). This suggests that *L. plantarum* has limited reliance on glutathione for oxidative stress management, likely compensating with alternative protective mechanisms such as NADH-dependent systems or manganese accumulation [73].

Overall, the data demonstrates that *L. plantarum* possesses a core probiotic genetic signature, characterized by universal GABA production and vitamin biosynthesis, widespread bile salt hydrolase activity, and moderate stress tolerance and antimicrobial potential. The radar plot (Figure 3) clearly illustrates these patterns, showing strong peaks for GABA production and vitamin biosynthesis, a secondary peak for bile salt tolerance, and smaller contributions from stress tolerance, adhesion, and bacteriocins. The near absence of glutathione metabolism is also evident. These results emphasize that while all strains share fundamental probiotic traits, the presence of accessory genes introduces strain-specific diversity, which is critical for tailoring *L. plantarum* strains for specialized probiotic and industrial applications.

### 3.4. Secondary Metabolite and Functional Potential

**Figure 4:**
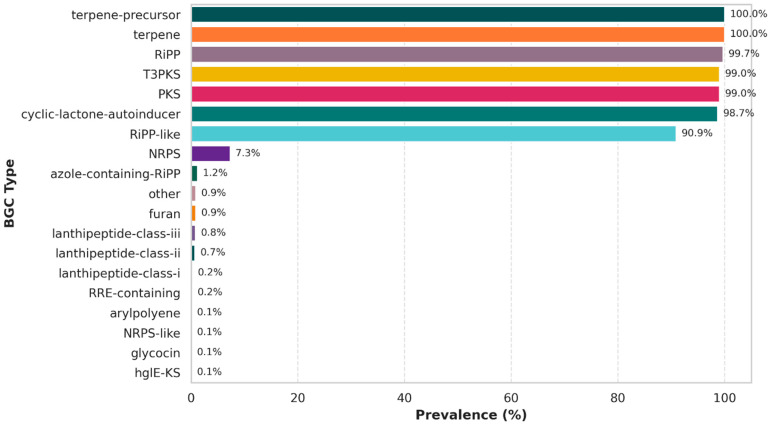
Horizontal bar plot showing the prevalence of BGC categories in *Lactiplantibacillus plantarum* (n = 1240 genomes). Terpene-precursor, terpene, RiPP, T3PKS, PKS, and cyclic-lactone-autoinducer clusters were nearly universal, while NRPS and specialized clusters (e.g., azole-containing RiPPs, lanthipeptides, glycocin, arylpolyene) occurred at low frequency, indicating a conserved metabolic core and sporadic accessory diversity.

Analysis of secondary metabolite BGCs across 1240 *L. plantarum* genomes revealed a highly conserved and diverse repertoire (Figure 4). The most prevalent clusters included terpene precursors and terpene synthases, which were detected in all genomes (100%). These clusters are associated with carotenoid- and isoprenoid-related metabolites, which may contribute to oxidative stress tolerance and potential health-promoting functions [74].

Ribosomally synthesized and post-translationally modified peptides (RiPPs) were also nearly ubiquitous, with canonical RiPP clusters detected in 99.7% of strains and RiPP-like clusters in ~91%. These findings suggest that RiPPs are a core feature of *L. plantarum*, possibly linked to bacteriocin-like antimicrobial activity that enhances ecological competitiveness in complex fermented food environments.

Additionally, Type III polyketide synthases (T3PKS) and PKS clusters were present in 99.0% of strains, indicating a strong genetic capacity for polyketide biosynthesis. While the exact metabolite products remain underexplored, these clusters may contribute to antioxidant activity and niche adaptation. Similarly, cyclic lactone autoinducer clusters were identified in 98.7% of genomes, supporting widespread potential for quorum-sensing–like signaling mechanisms.

In contrast, non-ribosomal peptide synthetase (NRPS) clusters were rare (~7.3%), and additional specialized clusters, including azole-containing RiPPs, lanthipeptides, glycocins, and arylpolyenes, occurred only sporadically (<1%). Azole-containing RiPPs and lanthipeptides are commonly associated with antimicrobial and bacteriocin-like activities, which have been reported to inhibit other pathogens [75]. Glycocins represent glycosylated bacteriocins that may confer enhanced stability and target specificity [76], and arylpolyenes have been linked to protective functions such as oxidative-stress resistance and membrane stabilization [77]. These low-frequency clusters constitute a small but potentially valuable reservoir of lineage-specific bioactivities that may contribute to strain-level functional differentiation.

Collectively, the radar of BGC distribution demonstrates that while *L. plantarum* carries a conserved core of terpenes, RiPPs, and polyketides; it also harbors a low-frequency accessory reservoir of rare clusters that may underpin strain-specific functional traits. This dual architecture—universal core BGCs complemented by sporadic accessory clusters—mirrors the species’ ecological versatility and probiotic potential [78,79].

### 3.5. Carbohydrate-Active Enzymes (CAZymes) in Lactiplantibacillus plantarum

Comprehensive CAZy annotation of 1240 *L. plantarum* genomes revealed a broad and diverse repertoire of carbohydrate-active enzymes (CAZymes), comprising glycoside hydrolases (GHs), glycosyltransferases (GTs), carbohydrate-binding modules (CBMs), carbohydrate esterases (CEs), and auxiliary activities (AAs). In total, 109 distinct CAZy families were identified.

**Figure 5:**
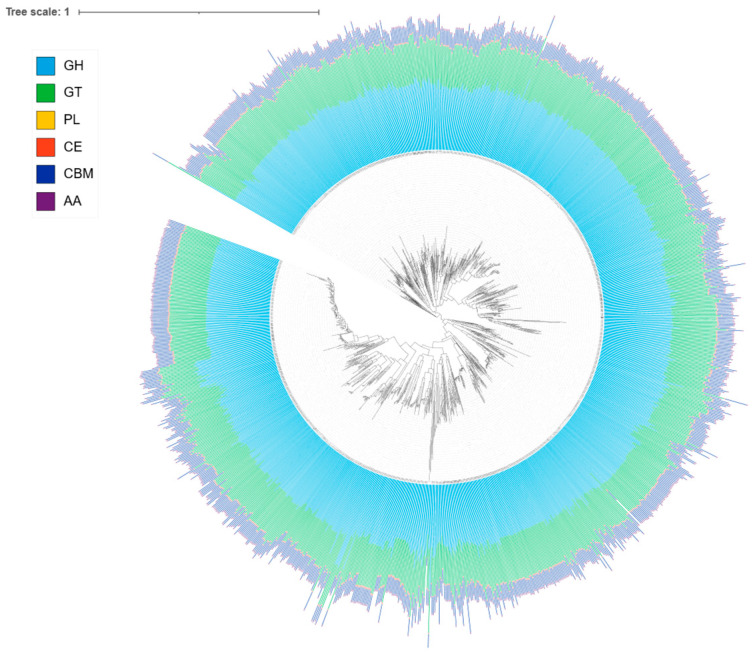
CAZy class composition per genome as a tip-aligned heatmap. For each strain, cells show the percentage of CAZyme annotations assigned to GH, GT, PL, CE, CBM, and AA. Darker shading indicates higher within-genome prevalence. The heatmap uses a single light-to-dark gradient for clarity and comparability across classes.

The most abundant families were GT4 (34,302 genes) and GT2 (33,296 genes), which contribute to polysaccharide biosynthesis and cell-wall assembly, as shown in Figure 5. Among GH families, GH1 (26,805 genes), GH13_31 (14,636), GH65 (12,319), GH25 (10,006), and GH23 (8837) were highly enriched, representing enzymes involved in starch degradation, β-glucoside hydrolysis, mannoside metabolism, and peptidoglycan turnover. Carbohydrate-binding modules such as CBM50 (18,122) and CBM48 (3526) were also prominent, reflecting enhanced substrate recognition and binding capabilities. Additionally, AA10 (3747 genes), associated with lytic polysaccharide monooxygenases, suggests oxidative capabilities in carbohydrate degradation.

At the category level, GHs dominated the dataset (143,570 genes; 52.5%), followed by GTs (89,749; 32.9%), CBMs (32,540; 11.9%), AAs (3747; 1.4%), and CEs (2590; 0.9%). Diversity analysis showed that individual genomes typically harbored 60–90 distinct CAZy families, indicating a conserved enzymatic backbone with strain-specific variation. Hierarchical clustering of presence/absence profiles further revealed that core families (e.g., GT2, GT4, GH1, GH13_31) were universally present across genomes, while families such as GH78, GH170, and certain CBMs appeared sporadically, highlighting functional specialization among strains.

The extensive CAZyme repertoire identified in *L. plantarum* underscores its metabolic flexibility and ecological versatility [80]. The predominance of GH families suggests that *L. plantarum* is well-equipped for degradation of a wide range of plant- and host-derived polysaccharides, facilitating survival in both environmental niches (e.g., fermented plant foods) and host-associated environments (e.g., the gastrointestinal tract) [81,82]. The consistent presence of GT families indicates robust capacity for cell wall biosynthesis and exopolysaccharide production, traits that contribute to stress tolerance, colonization, and probiotic potential [82].

High frequencies of CBM50 and CBM48 modules indicate strong substrate-binding capabilities, advantageous in nutrient-limited or plant-associated environments. The presence of AA10 enzymes further supports the ability to degrade recalcitrant carbohydrates such as chitin and cellulose [83,84].

Overall, the CAZyme landscape of *L. plantarum* reflects a dual strategy of hydrolysis and biosynthesis: hydrolysis allows for efficient nutrient acquisition from diverse carbohydrate sources, while biosynthesis (e.g., GT-mediated exopolysaccharides) enhances cell envelope functionality and may play roles in host interactions [80,84]. The observed strain-specific variability suggests potential for niche specialization, with some lineages possessing expanded CAZy repertoires that could confer selective advantages in particular fermentation substrates or host environments. These findings highlight *L. plantarum* as a highly versatile species with an enzymatic arsenal that not only underpins its success in diverse ecosystems but also supports its utility in food fermentation, probiotic applications, and functional carbohydrate biotransformation.

### 3.6. Pangenome Analysis

The pangenome was strongly open, with only 622 strict core genes (1.38%) and 444 soft-core genes (2.36%), compared to a vast accessory genome (97.64%). This reflects ecological diversity and frequent gene exchange via mobile elements [65]. Functionally, the accessory genome likely encodes the “tuning knobs” for substrate acquisition, stress/defense, and micro-niche adaptation, while a compact core preserves the conserved housekeeping and metabolic backbones.

**Figure 6:**
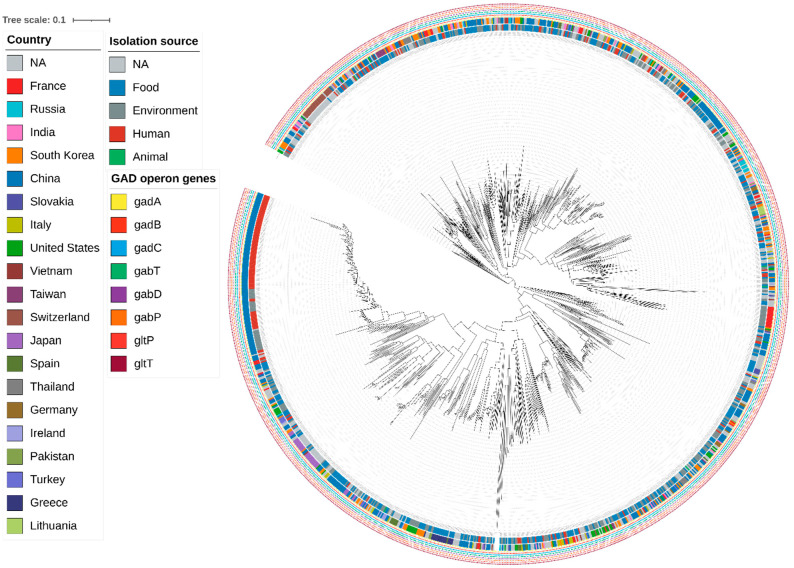
Accessory-genome phylogeny of 1240 *Lactiplantibacillus plantarum* genomes (iTOL). Tree inferred from a binary presence–absence matrix of accessory gene families, with branch lengths reflecting differences in accessory-gene repertoires. Concentric rings annotate: (1) *gad* operon (*gadB*/*gadC*) presence/absence, (2) country of isolation, and (3) isolation source category (Human, Animal, Food, Environment, NA). These overlays reveal clade-specific patterns of *gad* operon carriage and ecological/biogeographic structure. Figure rendered with iTOL (Interactive Tree of Life); color/category mappings follow the panel legend.

Despite this extensive accessory variation, the GABA operon (*gadB*, *gadC*) was universally conserved across all genomes and all isolation sources. Accessory-genome phylogeny revealed ecological and geographic clustering, but no operon loss, indicating that GABA biosynthesis is a species-level trait, as shown in Figure 6 [61,85].

At the strain level, phenotypes are driven chiefly by accessory genome content rather than the ubiquitous *gad* operon. Accordingly, when selecting strains for fermentation or probiotic applications targeting GABA production or acid stress tolerance, emphasis should be placed on accessory features—carbohydrate uptake/processing cassettes, bacteriocin/immunity loci, membrane- and stress-response modules, and plasmid-encoded functions—while treating *gadB*/*gadC* as baseline capabilities. This is supported by our complete-genome subset, in which plasmid load varies widely (median = 2; range = 0–17), highlighting the role of mobile elements in shaping niche-specific performance [85].

## 4. Conclusions

Across 1240 genomes, *L. plantarum* exhibits a highly open pangenome dominated by accessory loci, yet retains a universally conserved GABA operon (*gadB*, *gadC*), confirming GABA production as a core species capability. Strain-level phenotypes therefore arise primarily from accessory modules such as carbohydrate-utilization pathways, bacteriocin/immunity systems, stress-response functions, and plasmid-encoded traits, underscoring the role of mobile elements in niche-specific performance. The CAZy and BGC profiles further highlight a conserved metabolic backbone complemented by rare, lineage-specific functions. These findings provide a genomic framework for rational strain selection in fermentation and probiotic applications: treat *gadB*/*gadC* as baseline capabilities and prioritize accessory-encoded traits that align with the intended substrate, process conditions, and functional endpoints. These insights provide actionable criteria for rational strain selection in functional food innovation and probiotic product development.

## Figures and Tables

**Table 1:** Summary of GABA-related genes in *Lactiplantibacillus plantarum* (n = 1240).

Gene	Function	Number of Strains	Percentage (%)
*gadA*	Glutamate decarboxylase (GAD); catalyzes conversion of glutamate to GABA	0	0.00
*gadB*	Glutamate decarboxylase (GAD); catalyzes conversion of glutamate to GABA	1240	100.00
*gadC*	Glutamate/GABA antiporter; exports GABA in exchange for glutamate	1240	100.0
*gabT*	GABA aminotransferase; catabolizes GABA to succinate semialdehyde	0	0.00
*gabD*	Succinate semialdehyde dehydrogenase; converts SSA to succinate	3	0.24
*gabP*	GABA permease; imports GABA	0	0.00
*gltP*	Glutamate transporter (alternative system; not detected)	0	0.00
*gltT*	Glutamate transporter; supports glutamate uptake for GABA biosynthesis	1237	99.80

**Table 2:** Comparative prevalence (%) of GABA-related genes across Lactobacillus species.

Species	*gadA*	*gadB*	*gadC*	*gabT*	*gabD*	*gabP*	*gltP*	*gltT*
*L. brevis*	99.5	0.0	100.0	0.0	0.0	0.0	0.5	99.5
*L. buchneri*	57.4	0.0	100.0	0.0	0.0	0.0	98.1	98.1
*L. casei*	0.0	0.0	100.0	0.0	0.0	0.0	0.0	0.0
*L. fermentum*	40.8	0.0	100.0	0.0	99.8	0.0	0.7	99.1
*L. helveticus*	0.0	0.0	100.0	0.0	0.0	0.0	0.0	0.0
*L. paracasei*	0.2	0.0	100.0	0.0	1.4	0.0	0.0	0.0

**Table 3:** Reported GABA production by *Lactiplantibacillus plantarum* strains in previous studies.

Strain	Isolation Source	Reported GABA Production (g/L)	Genome Available	Year	Country	Reference
FNCC 260	Indonesian fermented foods	0.809	Yes	2021	Indonesia	[14]
FRT7	Chinese Paocai	1.159	No	2023	China	[12]
LSI2-1	Thai fermented vegetables	22.94	Yes	2021	Thailand	[9]
DW12	Thai fermented beverage (red seaweed)	Not specified	Yes	2021	Thailand	[47]
KB1253	Japanese pickle	Not specified	Yes	2019	Japan	[11]
NDC75017	Traditional fermented dairy products	3.15	No	2015	China	[48]
L10-11	Thai fermented fish (Plaa-som)	15.74	No	2021	Thailand	[49]
K154	Kimchi	15.53	No	2013	South Korea	[50]
EJ2014	Rice bran	19.80	No	2013	South Korea	[51]
C48	Cheese	0.016	No	2007	Italy	[15]
KCTC 3103	Unknown	0.67	No	–	–	[52]
Taj-Apis362	Honeycomb and honeybee stomach	0.737	No	2015	Malaysia	[53]
BC114	Sichuan Paocai (fermented vegetable)	3.45	No	–	China	[54]
MNZ	Fermented soybean	0.408	No	–	–	[55]
K255	Kimchi	0.821	No	–	South Korea	PubMed
SPS109	Thai fermented food	1.157	Yes	2024	Thailand	[56]

**Table 4:** Probiotic-Related Genes in *Lactiplantibacillus plantarum* (1240 genomes). Genes are grouped by functional category, including acid tolerance and GABA metabolism, bile salt hydrolase activity, stress tolerance, adhesion and surface-associated functions, bacteriocin production, vitamin biosynthesis, and glutathione metabolism. Prevalence values indicate the percentage of genomes in which each gene was detected.

Category	Gene	Function/Role	Prevalence (%)
Acid tolerance/GABA	*gadB*	Glutamate decarboxylase A—converts glutamate to GABA (acid tolerance, neurotransmitter production)	100.0
	*gltT*	Glutamate transporter—glutamate uptake	99.8
	*gadC*	Glutamate/GABA antiporter—exports GABA, imports glutamate	100.0
	*gabD*	Succinic semialdehyde dehydrogenase—GABA catabolism	0.2
	*gabT*	GABA transaminase—GABA shunt metabolism	0.0
	*gltP*	Proton/glutamate symporter—glutamate uptake	0.0
Bile salt hydrolase	*bsh*	Bile salt hydrolase—detoxifies bile salts, enhances gut survival	88.9
Stress tolerance	*dnaK*	Heat shock protein Hsp70—stress response, protein folding	37.7
	*groEL*	Chaperonin—protein folding under stress	56.9
Adhesion and surface	*slpA*	Surface layer protein—adhesion to host cells	25.0
Bacteriocins	*plnE*	Plantaricin E—antimicrobial peptide	47.7
	*plnF*	Plantaricin F—antimicrobial peptide	46.5
	*pediocin*	Pediocin-like bacteriocin—antimicrobial against pathogens	1.0
Vitamin biosynthesis	*folA*	Dihydrofolate reductase—folate biosynthesis	100.0
	*folB*	Dihydroneopterin aldolase—folate biosynthesis	100.0
	*folC*	Folate synthase—folate biosynthesis	100.0
	*ribA*	GTP cyclohydrolase II—riboflavin biosynthesis	100.0
	*ribB*	3,4-dihydroxy-2-butanone-4-phosphate synthase—riboflavin biosynthesis	100.0
	*ribC*	Riboflavin synthase subunit—riboflavin biosynthesis	100.0
	*ribD*	Diaminohydroxyphosphoribosylaminopyrimidine deaminase—riboflavin biosynthesis	100.0
	*ribE*	Lumazine synthase—riboflavin biosynthesis	100.0
Glutathione metabolism	*gshA*	Glutamate–cysteine ligase—glutathione biosynthesis	0.2
	*gshB*	Glutathione synthetase—glutathione biosynthesis	0.2

## Data Availability

All genome sequences analyzed in this study were obtained from publicly available datasets in the NCBI database.

## Supplementary Materials

The following supporting information can be downloaded at: https://www.mdpi.com/article/10.3390/life16010047/s1, Table S1. Metadata of downloaded genomes from NCBI database.
